# Targeting matrix metalloproteases in diabetic wound healing

**DOI:** 10.3389/fimmu.2023.1089001

**Published:** 2023-02-17

**Authors:** Junren Chen, Siqi Qin, Shengmeng Liu, Kexin Zhong, Yiqi Jing, Xuan Wu, Fu Peng, Dan Li, Cheng Peng

**Affiliations:** ^1^ State Key Laboratory of Southwestern Chinese Medicine Resources, School of Pharmacy, Chengdu University of Traditional Chinese Medicine, Chengdu, China; ^2^ State Key Laboratory of Quality Research in Chinese Medicine, Macau Institute for Applied Research in Medicine and Health, Macau University of Science and Technology, Taipa, Macau SAR, China; ^3^ Key Laboratory of Drug-Targeting and Drug Delivery System of the Education Ministry, Department of Pharmacology, Sichuan University, Chengdu, China; ^4^ Sichuan Engineering Laboratory for Plant-Sourced Drug and Sichuan Research Center for Drug Precision Industrial Technology, West China School of Pharmacy, Sichuan University, Chengdu, China

**Keywords:** matrix metalloproteases, diabetic wound healing, natural products, chronic inflammation, clinical research studies

## Abstract

Chronic inflammation participates in the progression of multiple chronic diseases, including obesity, diabetes mellitus (DM), and DM related complications. Diabetic ulcer, characterized by chronic wounds that are recalcitrant to healing, is a serious complication of DM tremendously affecting the quality of life of patients and imposing a costly medical burden on society. Matrix metalloproteases (MMPs) are a family of zinc endopeptidases with the capacity of degrading all the components of the extracellular matrix, which play a pivotal part in healing process under various conditions including DM. During diabetic wound healing, the dynamic changes of MMPs in the serum, skin tissues, and wound fluid of patients are in connection with the degree of wound recovery, suggesting that MMPs can function as essential biomarkers for the diagnosis of diabetic ulcer. MMPs participate in various biological processes relevant to diabetic ulcer, such as ECM secretion, granulation tissue configuration, angiogenesis, collagen growth, re-epithelization, inflammatory response, as well as oxidative stress, thus, seeking and developing agents targeting MMPs has emerged as a potential way to treat diabetic ulcer. Natural products especially flavonoids, polysaccharides, alkaloids, polypeptides, and estrogens extracted from herbs, vegetables, as well as animals that have been extensively illustrated to treat diabetic ulcer through targeting MMPs-mediated signaling pathways, are discussed in this review and may contribute to the development of functional foods or drug candidates for diabetic ulcer therapy. This review highlights the regulation of MMPs in diabetic wound healing, and the potential therapeutic ability of natural products for diabetic wound healing by targeting MMPs.

## 1 Introduction

Diabetes mellitus (DM) is a chronic metabolic noncommunicable disease principally characterized by consistently high blood glucose levels that may affect more than 783 million individuals in 2045 worldwide (1, 2). Sustained exposure to high levels of glucose induces neurological, microvascular and macrovascular lesions and low immune response in the body, which thereby contributes to the impaired wound healing, a crucial matter in diabetic patients (3). Such chronic wound ulcer is often exacerbated by tissue ischemia or constant stress, especially in the foot, which can eventually lead to amputation if no appropriate therapeutic strategy are applied (4). Besides, high glucose (HG) also affects the functions of cornea, leading to several diabetic corneal complications especially delayed epithelial wound healing (5). Unlike wound healing in healthy individuals, the healing process in DM is retarded in the inflammatory phase, as manifested by the elevation of pro-inflammatory cytokines, proteases, and reactive oxygen species (ROS), and the dysfunctions of numerous cell types (6). In addition, wounds under diabetic condition are more susceptible to bacterial infection on account of damaged immune responses, which brings about substantial recruitment of inflammatory cells that produce various ROS and destroy structural elements of the extracellular matrix (ECM) (7). Noticeably, ROS together with pro-inflammatory cytokines can further impairs the wound by inducing matrix metalloproteinases (MMPs) expression, which results in degradation of the ECM and growth factors (8).

MMPs, a set of zinc-dependent proteolytic enzymes, participate in kinds of wound healing events by degrading almost all protein components of ECM (9). According to the structure of substrates and domains, MMPs can be primarily classified as collagenases (like MMP-1, MMP-8, and MMP-13), matrilysins (such as MMP-7), stromelysins (such as MMP-3, MMP-10, and MMP-11), gelatinases (such as MMP-2 and MMP-9), membrane type metalloproteinases (such as MMP-14), as well as others (10, 11). Usually, MMPs are inactive and exist as latent precursors “zymogens” *in vivo*, but turn into active status when stimulated by external stimuli like cytokines, growth factors, as well as cell-matrix interactions (12). A critical mechanism for the modulation of MMPs is through conjugation with endogenous inhibitors, such as tissue inhibitors of metalloproteinases (TIMPs), α2-macroglobulin, and small molecules with TIMP-like domains (13–15). During the progress of diabetic ulcer, MMPs show higher level of protease activity, they degrade protein, undermine the temporary ECM, remodel the granulation tissue, modulate angiogenesis, and administrate the activity of growth factors (16–18). Intriguingly, multiple signaling pathways associated with inflammatory response, oxidative stress, and apoptosis, as well as epigenetic modification are illustrated to be implicated in the regulation of MMPs during diabetic wound healing (19–21), which indicate that targeting these signals-mediated MMPs is a promising strategy for diabetic ulcer.

Natural products are a dominating resource for developing drug candidates with specific bioactivities to prevent diseases and can function as dietary supplements to provide benefits for human health. Over the past decade, increasing evidence has shown that naturally occurring flavonoids, polysaccharides, saponins, and alkaloids exhibit outstanding therapeutic activities on wound healing under a variety of pathological conditions (22). In addition, animal-derived peptides and hormones have also been reported to possess promising ability to improve diabetic wound healing (23, 24). These compounds mainly improve diabetic wound healing through accelerating re-epithelization and ECM formation, alleviating oxidative stress, relieving inflammation, promoting angiogenesis, and suppressing apoptosis *via* MMPs-relevant signaling pathways (25–29). As a result of the favorable ability of natural products in regulating aberrantly altered MMPs during diabetic wound healing, developing the novel agents based on the structures of these natural products may contribute to the treatment or mitigation of diabetic ulcers.

This review highlights the physiological and pathological regulation of MMPs in diabetic ulcers, and the potential therapeutic ability as well as mechanisms of natural products for diabetic wound healing by targeting MMPs. In addition, clinical research studies focused on MMPs in the process of diabetic wound healing are also addressed. At the same time, the application prospect of natural products targeting MMP in the prevention and treatment of diabetic ulcers is pointed.

## 2 MMPs in diabetic ulcer

### 2.1 Stem cells and MMPs

Mesenchymal stem cells (MSCs) are illustrated to accelerate wound healing under diabetic condition *via* regulating ECM proteolysis. MSCs reverses the reduction of COL I and COL II, down-regulates MMP-9 expression, and suppresses the levels of activated MMP-9 in diabetic wounds. In addition, MSCs administration up-regulates the expression of miR-29b in diabetic wounds and HG-treated dermal fibroblasts (30), indicating that MSCs exert therapeutic effect on diabetic wound healing through repressing proteolysis and improving COL levels in ECM *via* targeting miR-29/MMP-9 axis. Interestingly, adipose derived mesenchymal stem cells (ADSCs) are demonstrated to improve diabetic ulcer through modulating the expression of ECM remodeling-associated genes. Ghaneialvar et al. reported that ADSCs administration down-regulates the gene expressions of MMP-9 and up-regulates the expressions of MMP-2 as well as TIMP-1 in STZ-induced mice. Besides, the mRNA expression of urokinase-type plasminogen activator (uPA) is elevated at the early stages of wound healing process, which may promote the activity of MMP in the inflammatory phase, while ADSCs intervention reduces the expression of uPA in diabetic group (31). Additionally, Wang et al. reported that extracellular vesicles derived from adipose-derived stem cells (ADSC-EVs) play a significant role in diabetic wound healing by promoting collagen (COL) deposition through down-regulating MMP-9 expression. *In vitro* studies demonstrated that ADSC-EVs promotes the proliferation of AGEs-BSA-treated HaCaT cells, while inhibits the secretion of MMP-9. Besides, ADSC-EVs improves the wound healing rate of diabetic mice through accelerating the re-epithelialisation, facilitating COL deposition, and reducing MMP-9 levels in the wound fluids (32).

Bone-marrow-derived mesenchymal stem cells (BM-MSCs) has been utilized as effective therapeutic strategy for wound healing due to their abilities to modulated inflammation, ECM production, as well as angiogenesis. Kamiya et al. reported that BM-MSCs transplantation accelerates wound healing in STZ-induced rats and HG-induced keratinocytes through improving re-epithelialization, elevating the suppressed viability of HKCs, and increasing the expressions of MMP-2, epidermal growth factor (EGF), human insulin-like growth factor 1 (IGF-1) as well as p-FAK (33). In addition, mouse bone marrow (BM)-derived allogeneic MSCs (allo-mBM-MSCs) facilitate wound healing in STZ-induced diabetic mice through secreting the growth factors and proteins such as MMP-1. Allo-mBM-MSCs administration improves the wound healing rate through promoting the re-epithelialization, granulation tissue formation, COL deposition and vascular proliferation. Notably, Allo-mBM-MSCs accelerates the wound repair through secreting the factors and proteins such as COL-1, keratinocyte growth factor, MMP-1, Ang-2, IGF-1, hepatocyte growth factor, prostaglandin E2 and vascular endothelial growth factor (VEGF) (34). Altogether, these results suggest that modulating the secretion of MMP-1 by allo-mBM-MSCs may be a potential therapy for diabetic wound repair.

Stem cells from human exfoliated deciduous teeth (SHED) possess strong differentiation capacity that display outstanding therapeutic effect in wound repair. Lv et al. found that SHED treatment up-regulates MMP-9 and MMP-2 in rats with DFU, resulting in the improvement of wound healing, enhancement of angiogenesis, reduction of inflammation, as evidenced by the up-regulation of VEGF and eNOS, as well as the down-regulation of interleukin (IL)-10, Tumor necrosis factor (TNF)-1α as well as IL-1β. However, SHED transplantation is not as effective as MSCs in wound healing (35). Even so, SHED is a potential treatment for diabetic ulcer healing and may address the surgical invasiveness associated with MSCs transplantation.

Considerable studies have illustrated that endothelial progenitor cells (EPCs) play a pivotal part in vasculo-genesis, which thereby gives rise to the reconstitution of microcirculation and healing. Impeded neovascularization and impaired EPCs are major features of diabetic wound healing. Angiopoietin (Ang)-1is a potent mobilizer of EPCs from the BM, which improves re-epithelialization and EPC recruitment in the wounds of diabetic mice though up-regulating the expression of MMP-9 and stem cell factor (SCF). Interestingly, SCF treatment can reverses the decreased mobilization of EPCs in MMP-9^-/-^ mice, and Ang-1 overexpression elevates the re-epithelialization of wounds in MMP-9^-/-^ mice (36), which suggest that the protective effects of Ang-1 on diabetic wound healing are involved in the EPC recruitment and MMP-9 regulation.

### 2.2 Inflammation-associated signals pathways and MMPs

During the development of diabetic wounds, the excessive inflammation coupled with advanced glycation end products (AGEs) accumulation cause down-regulation of growth factors, rapid degradation of matrix, as well as reduction of COL, ultimately leading to impeded wound healing in patients (37). Numerous studies have reported that nuclear transcription factor-κB (NF-κB) signaling pathways contribute to the expression of MMPs in diabetic foot ulcers (DFUs). Chang et al. reported that DFUs infection impairs the wound healing by increasing inflammation and inhibiting angiogenesis, which gives rise to the up-regulation of MMP-9 through activating the NF-κB signaling pathway *via* increasing ROS, whereas it does not affect the level of MMP-8. Remarkably, the inhibitor of MMP-8 delays the diabetic wound healing, but (R)-ND-336, the inhibitor of MMP-9, promotes the wound repair, which suggest that MMP-8 facilitates wound healing in DM, whereas MMP-9 does not (18, 38). In addition, Notch1/NF-κB/MMP-9 axis also participates in diabetic wound closure. Zhu et al. reported that Notch1 signaling pathway is activated in skin of diabetic rats and AGEs-BSA-treated primary human keratinocytes, as evidenced by the up-regulation of Notch intracellular domain (NICD), Delta-like 4 (Dll4), as well as Hes1, which contributes to the elevation of MMP-9 activation. Remarkably, the regulatory effect of Notch1 on MMP-9 relies on NF-κB activation, and suppression of Notch1 significantly prevents the nuclear translocation of NF-κB induced by AGEs-BSA in keratinocytes. Interestingly, inhibiting Notch1 signal with DAPT represses NICD and MMP-9, resulting in the improvement of COL accumulation and diabetic wound healing (39). Moreover, MMP-9 in diabetic wound healing is also mediated by receptor for AGE (RAGE), MAPK as well as NF-κB signaling pathways. In AGEs-BSA-treated HaCaT cells, MMP-9 expressions are significantly up-regulated, while such effect is reversed by the intervention of inhibitors of extracellular regulated protein kinases (ERK)1/2, p38, as well as NF-κB. In addition, AGEs-BSA also elevates the expression of RAGE in HaCaT cells and promotes NF-κB p65 translocation. Remarkably, silence of RAGE abrogates MMP-9 activation and the phosphorylation of ERK1/2 as well as p38 (21).

Indeed, long-term HG exposure is elucidated to impair keratinocyte migration and obstacle wound healing through stimulating M1 macrophage polarization *via* TNF-α TIMP-1/MMP-1 axis. Huang et al. reported that pro-inflammatory M1 macrophages and TNF-α levels are obviously increased in the perilesional area of diabetic rats, as evidenced by the up-regulated ratio of C-C chemokine receptor type 7 (CCR7)/CD68. Besides, TNF-α from M1 type macrophage suppresses the migration of keratinocytes by down-regulating MMP-1 and up-regulating TIMP-1, while TNF-α antibody addition or gene-silencing of TIMP-1 restore the impaired function of keratinocytes. Further *in vivo* studies demonstrated that TNF-α antagonist promotes wound healing process in diabetic rats (40). In addition to high systemic blood glucose concentration, local hyperglycemia can also inhibit wound healing. Kruse et al. found that the migration of keratinocyte and fibroblast is suppressed by 5.6 mM glucose intervention, which results in the delayed close of scratch wounds. In addition, local hyperglycemia inhibits the wound healing and re-epithelialization in rats through increasing the levels MMP-1 (41). Interestingly, Feng et al. reported that MMP-9 blocks the wound healing process in DM mice through attenuating EPCs recruitment *via* suppressing CXCL12 activation. The expressions of phagocyte-derived MMP-9 and pro-inflammation factors (such as TNF-α and IL-6) are augmented, whereas the numbers of EPC and levels of CXCL12 are decreased in STZ-induced diabetic mouse. However, the inhibitor of MMP significantly facilities the diabetic wound healing compared with TNFα-treated group (42). Therefore, these results suggest that the inhibitor of MMPs may be a potential agent for impaired wound closure in diabetic patients. Furthermore, IL-1β is demonstrated to impede fibroblasts functions from diabetic wound tissues by modulating the expression of MMPs *via* a p38-mediated pathway. A recent study reported that IL-1β levels are up-regulated in the wounds and serum of diabetic individuals as well as that of *db/db* mice, which suppresses the proliferation and migration of fibroblasts, enhances MMP-2 and MMP-9 expression, and down-regulates TIMP-1 and TIMP-2. Additionally, IL-1β intervention dose-dependently promotes the phosphorylation of p38 in cultured fibroblasts, while SB203580 (a p38 inhibitor) countervails the effects of IL-1β on collagenase, MMPs, and TIMPs (43), suggesting that IL-1β takes part in delayed wound healing in DM by altering levels of ECM remodeling proteins through activating p38 signal.

**Figure 1:**
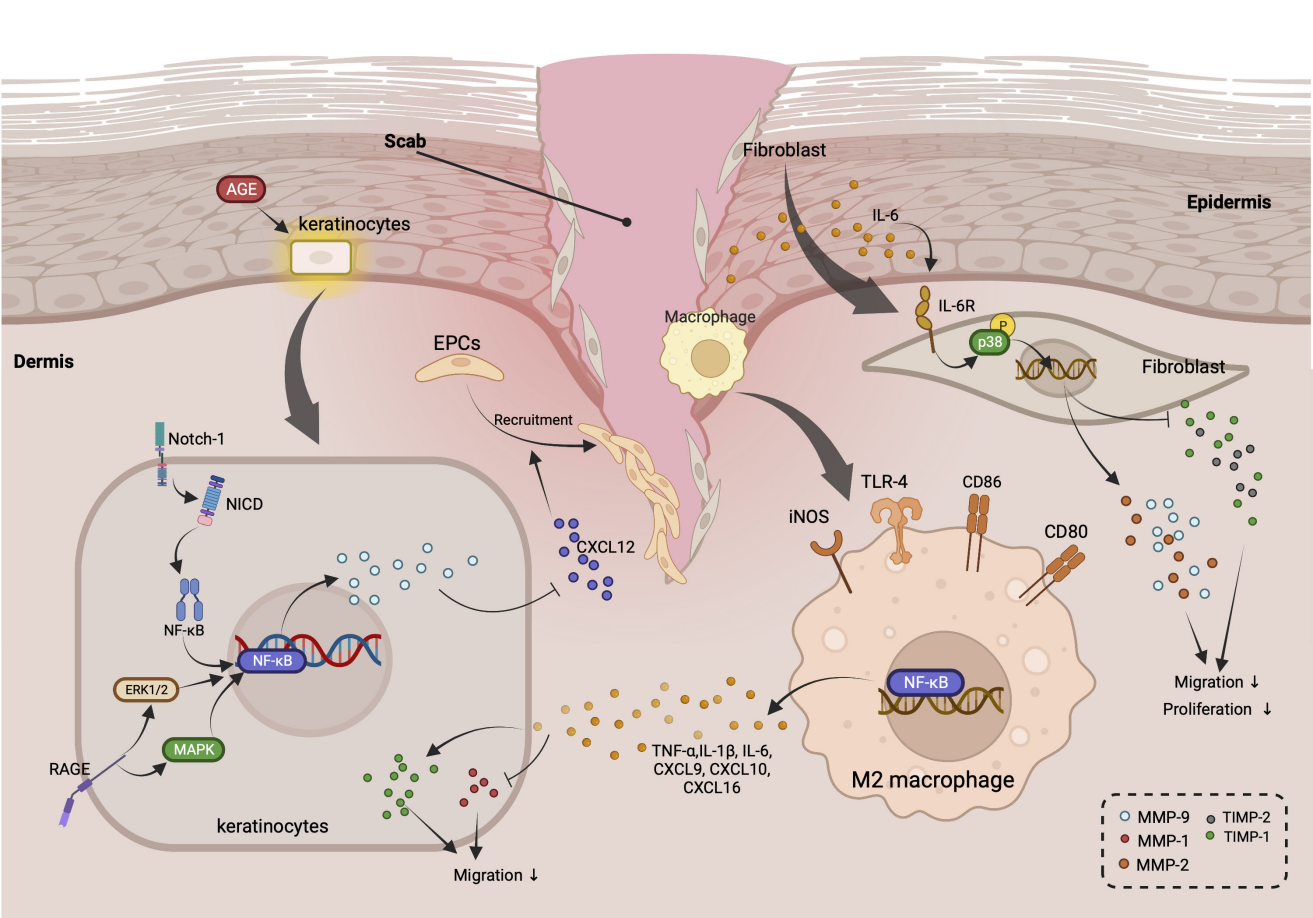
Roles of MMPs in regulating inflammation-related cross-talks among keratinocytes, macrophage, and fibroblast during diabetic wound healing. (AGEs stimulates the activation of Notch-1/NICD/NF-κB, RAGE/ERK1/2, and MAPK signaling pathways in keratinocytes, which gives rise to the up-regulation of MMP-9. MMP-9 released by keratinocytes suppresses the recruitment of EPCs through down-regulating the expression of CXCL-12. In addition, TNF-α, IL-6, and other pro-inflammatory cytokines secretes by M2 macrophage elevate TIMP-1 and reduce of MMP-1, resulting in the suppressed migration of keratinocytes. Intriguingly, IL-6 also inhibits the migration as well as proliferation of fibroblast *via* promoting the expression of MMP-2 and MMP-9, while decreasing the levels of TIMP-2 through activating IL-6R/p38 pathway.).

Altogether, these studies indicate that targeting inflammatory response-relevant signaling pathways involving NF-κB, MAPK, TNF-α, as well as IL-1β is a promising therapeutic strategy to modulate MMP-9, MMP-8, and MMP-1 expression during diabetic ulceration (**Figure 1**).

### 2.3 Oxidative stress-associated signals and MMPs

ERK1/2 signal is demonstrated to participate the development of diabetic wound healing by modulating MMPs and activator protein-1(AP-1). AP-1 comprises c-Jun and c-Fos proteins and has been illustrated to function as the modulator of MMPs transcriptions under the diabetic condition. The protein levels of AP-1 and MMP-9 are enhanced in the epithelium of diabetic skin tissues. Besides, the protein stability of c-FOS/c-Jun, the subunits of AP-1, as well as the activation of ERK1/2 are elevated in HG-treated HaCaT cells, while ERK1/2 inhibitor reverses the phosphorylation of c-FOS and c-Jun, and down-regulates MMP-9 expression, suggesting that ERK1/2 activated by HG can stabilize AP-1, which leads to the transcription and expression of MMP-9 and subsequently the delayed wound healing (44). In addition, increasing studies proved that the transcriptions of MMP-2 as well as MMP-9 are regulated by AP-1, and c-Jun is a suppresser of p53 in immortalized fibroblasts. Tombulturk et al. reported that c-Jun, MMP-2, as well as MMP-9 are up-regulated in diabetic rats during wound healing process concomitant with the increase of p53 (20). Moreover, several up-stream signals have been considered as important targets for ERK1/2-mediated MMPs regulation in diabetic ulcer. CXCL16-CXCR6 axis promotes the diabetic wound healing in diabetic mice and *db/db* mice through improving MSC migration by targeting the expression of MMP-2 *via* FAK-Src-ERK1/2-MMP2 signaling pathway. Dhoke et al. observed that CXCR6 gene therapy facilitates the wound healing in mice with T1DM or T2DM through accelerating the re-epithelialization and neovascularization. Meanwhile, CXCR6 overexpression up-regulates the expression of MMP-2 through increasing the levels of FAK, Src and ERK1/2 *in vitro* experiment. Interestingly, the inhibitor of CXCL16 or the lack of CXCR6 gene attenuates wound repair through inhibiting the MSC migration and the increase of MMP-2, which suggests that CXCL16-CXCR6 axis play a critical role in diabetic wound recovery (45). In a nutshell, these findings reveal that interfering with ERK1/2-centered signaling pathways is paramount for regulating the expression of MMPs in diabetic wound healing.

Nuclear factor erythroid 2-related factor 2 (Nrf-2) regulates the adaptive response to exogenous and endogenous oxidative stresses. Previous studies have shown that severe oxidative stress can be observed in the wound tissue of DM patients, as revealed by activation of Nrf-2 as well as the downstream genes such as HO-1 and NQO1. Long et al. reported that Nrf2^-/-^ diabetic mice exhibits poor wound healing, which is due to oxidative DNA damage, up-regulation of MMP-9, and decrees of transforming growth factor (TGF)-β1. Nevertheless, Nrf-2 activation contributes to wound healing in HaCaT cells, which is conferred through elevating TGF-β1 and suppressing MMP-9 (46). Thus, it suggests that targeting NRf-2/MMP-9 is a promising axis for treating diabetic wound healing. Furthermore, elevated secretion of ROS in diabetic wounds is regarded as a hazardous factor that may contribute to delayed wound healing under the diabetic condition through prolonging infiltration of M1 macrophages and impairing dermal fibroblast and keratinocyte function (47). Seraphim et al. found that higher M1/M2 macrophage ratio and basal ROS levels, as well as decreased antioxidant defenses and angiogenesis are observed in Rag-2 and IL-2Rγ double knockout (KO) diabetic mice that lack T, B, as well as innate lymphoid cells cell function. However, the increased expression of MMP-9 in diabetic conditions is not observed in KO mice, which demonstrated that lymphocyte may mediate the up-regulation of MMP-9 in diabetic wounds to a certain degree (48). However, such mechanism is not clear at present, and further experiments are needed to verify how lymphocyte affects the expression of MMP-9 under diabetic condition.

### 2.4 Apoptosis-related signals and MMPs

Apoptosis has been illustrated to plays a pivotal part in diabetic ulcer, and recent studies reported that MMP-9 contributes to delayed wound healing under diabetic condition through regulating fibroblasts apoptosis, while TIMP-1 is demonstrated to promote cells growth and prevent apoptosis. Down-regulated expression of TIMP-1 can be observed in diabetic skin tissues as well as in AGEs-intervened fibroblasts, whereas active protein of TIMP-1 prevents apoptosis triggered by AGEs or DM (49). MMP-9 is significantly up-regulated in HG and hyper-homocysteine medium-treated skin fibroblasts, which results in the decrease of cell proliferation, viability, COL secretion, and migration. However, these inhibitory effects of MMP-9 on fibroblasts are abrogated by TIMP-1 (50). Moreover, AGEs-BSA suppresses the migration of keratinocyte through increasing MMP-9 expression, while reducing TIMP-1 levels. Besides, AGEs-BSA application also down-regulates the expression of phospho-focal adhesion kinase- Tyr397 (p-FAK) as well as α2β1 in keratinocytes (51), suggesting that in the context of the chronic hyperglycemia, the influences of AGEs-BSA on keratinocyte are conferred through regulating MMP-9/TIMP-1, p-FAK, and α2β1 integrin. Besides, Yang et al. observed that MMP-9 and TIMP-1 levels in diabetic rats changed dynamically with the alteration of wound. Specifically, the mRNA and protein levels of MMP-9 are obviously elevated in DM rats compared with control group, which reach the peak on day 3. On contrary, the mRNA and protein levels of TIMP-1 are obviously lowered, leading to the increase of MMP-9/TIMP-1 ratio. Intriguingly, changes in MMP-9 and TIMP-1 levels occur long before the skin is traumatized, suggesting the presence of hidden damage to diabetic skin that may cause ulcers (52). Thus, it suggests that targeting MMP-9/TIMP-1 axis is a promising strategy to alleviate apoptosis during diabetic wound healing.

Additionally, a recent study manifested that FasL/Fas signal is also implicated in the regulation of MMP in diabetic wound ulcer. Elevated MMP-9 in AGEs-treated keratinocytes promotes the apoptosis of keratin-forming cells through up-regulating the expression levels of cleaved caspases-3 as well as FasL, which suggests that MMP-9 may exert pro-apoptotic effect to suppress diabetic wound healing *via* a FasL/Fas-mediated pathway (53). Thus, targeting MMP-9/FasL/Fas axis may be a feasible strategy for diabetic wound healing therapy.

### 2.5 Non-coding RNAs and MMPs

Epigenetic modification especially the regulation of non-coding RNAs like microRNAs (miRNAs), long non-coding RNAs (lncRNAs), circular RNAs (circ_ RNAs), as well as small hairpin RNA (shRNA) on the secretion and expression of MMPs, has been considered as another integral mechanism associated with the pathogenesis of diabetic ulcer. Wang et al. observed that excessive miR-129 and miR-335 down-regulate the expression of MMP-9 *via* directly targeting Sp1 in AGEs-treated HaCaT cells. *In vivo* studies illustrated that miR-129 and miR-335 agomir accelerates wound healing through improving re-epithelialization and COL deposition by decreasing Sp1 and MMP-9 in diabetic rats, which suggest that miR-129 and miR-335 regulate MMP-9 levels *via* Sp1-mediated axis (54). In addition, miR-21 is demonstrated to promote wound healing in DM rats through improving fibroblast functions *via* targeting MMPs. Human keratinocyte-derived microvesicle miRNA-21 increases the migration as well as fibroblast-mediated angiogenesis, and accelerates diabetic cutaneous wound healing in rats through up-regulating IL-6, IL-8, MMP-1, as well as MMP-3, down-regulating TIMP-3 and TIMP-4, suppressing the expression of PTEN and RECK, and activating MAPK/ERK signaling pathway (55). Besides, miR-217 also participates in diabetic wound healing *via* modulating hypoxia inducible factor-1α (HIF‐1α)/VEGF pathway and the down-stream proteins such as MMP-2 and MMP-9. The serum levels of miR-217 are enhanced in DFU individuals and rats, which causes the down-regulation of VEGF by repressing the target gene HIF-1α. Noticeable, inhibition of miR-217 reduces foot ulcer area, improves ulcer healing, and elevates the micro-vessel density through suppressing the levels of inflammatory factors, while up-regulating MMP-2, MMP-9, VEGF, VEGFR-2, and eNOS in DFU rats (56).

Moreover, ten-eleven translocation-2 (TET2)-interacting lncRNA (TETILA) can facilitate active DNA demethylation of the MMP-9 promoter in wound healing under diabetic condition. TETILA is obviously elevated in HaCaT cells and diabetic skin tissues induced by AGEs, which enhances the protein levels of TET2 as well as its nuclear translocation, thus activating MMP-9 promoter demethylation. Besides, as a molecular scaffold, TETILA provides a binding surface for the assemble of TET2 and thymine-DNA glycosylase (TDG), contributing to the base excision repair-mediated MMP-9 promoter demethylation and the transcriptional activation of MMP-9 (19). Thus, it suggests that TETILA may function as a genomic homing signal for TET2-mediated demethylation specific loci in MMP-9 promoter, which ultimately disrupts the progress of wound healing in DM. Additionally, Circ_PRKDC is illustrated to hamper wound healing in DFUs by modulating the proliferation and migration of keratinocyte. Circ_PRKDC overexpression down-regulates MMP-2 and MMP-9 in human epidermal keratinocytes through targeting miR-31/FBN1 axis, which results in the suppression of cell migration (57). Notably, the slow wound healing of diabetic cornea is related to MMP-10 overexpression. Studies show that recombinant adenovire-driven shRNA promotes wound healing in diabetic corneas by inhibiting MMP-10 and cathepsin F, which activates the phosphorylation of epidermal growth factor receptor (EGFR) and Akt. Also, the combination of shRNA and c-Met overexpression can activate p38 and thus the downstream EGFR-Akt pathway, showing a more significant wound healing effect (58), suggesting that targeting MMP-10/EGFR/Akt is a promising axis for the treatment of diabetic keratopathy. Taken together, these studies manifest that targeting non-coding RNAs-mediated signaling pathways is a feasible option to modulate the expression of MMPs in wound healing under the diabetic condition.

### 2.6 FOXO-1 and MMPs

Recently, increasing evidences illustrated that forkhead box protein O1(FOXO1) is a critical regulator in wound healing, whose up-regulation may lead to the deterioration of diabetic ulcer. Foxo1^L/L^ diabetic mice exhibits better wound healing, which is related to down-regulation of MMP-9 and decrees of FOXO1. Remarkably, elevation of FOXO1 in HG-treated keratinocytes enhances the transcriptional activity as well as expression of MMP-9 through binding to its promoter, whereas FOXO1 depletion prevents HG-induced keratinocyte migration, through up-regulating the expression of the TIMP1 while inhibiting the expression of MMP-9 (59). Thus, it suggests that targeting FOXO1/MMP-9 axis is a possible way for the treatment of diabetic wound healing.

### 2.7 uPA/uPAR and MMPs

Recent studies found that corneal wounds of diabetic mice heals more slowly than those of normal mice, which may be related to the inhibition of Serpine1(PAI-1), uPA, and uPA receptor (uPAR) expression by hyperglycemia. Interestingly, epithelial wound healing is accelerated by the addition of Serpine1 to the corneal conjunctiva of diabetic mice. Further experiments show that increasing Serpine1 up-regrates the expression of Plau, Plaur and MMP-3 in the cornea of DM mice (60), which indicates that targeting uPA proteolytic pathway is a promising option for regulating MMP-3 in treating diabetic keratopathy. Transcription factor homeobox A3 (HOXA3) plays a principal part in wound repair and angiogenesis, which is increased during wound healing and leads to the elevation of endothelial cell migration, promotion of angiogenesis, and up-regulation the levels of MMP-14 as well as uPAR in endothelial cells. Nevertheless, the expression of HOXA3 is blocked in the wounds of diabetic mice, contributing to the delayed wound repair and angiogenesis. Exogenous HOXA3 application reverses these adverse phenomena in diabetic mice, and facilitates migration of endothelial cells and keratinocytes *via* a uPAR-dependent mechanism (61). In berif, targeting uPA/uPAR is a possible way to modulate MMPs expression in diabetic wound healing.

### 2.8 DNA methylation and MMPs

Site-specific DNA demethylation of the MMP-9 promoter is demonstrated to be a paramount mechanism for MMP-9 regulation during diabetic wound healing. Ling et al. reported that TNF-α intervention augments MMP-9 expression and decrease the demethylation trend at the -36 bp promoter site in HaCaT cells. Besides, the alteration at the -36 bp site is the most significant among the CpG sites that distinctively demethylated in the MMP-9 promoter region, and higher transcriptional activity can be detected in the promoter with only the -36 bp site demethylated, which suggests that the -36 bp site is required in MMP-9 expression, while other CpG sites might play synergistic effects in TNF-α-stimulated keratinocytes (62). In addition, the activation of MMP-9 in AGEs-BAS-induced HaCaT cells is accompanied by the elevation of RhoA, GTP-RhoA and ROCK1, suggesting that mevalonate pathway participates in the expression of MMP-9 in AGEs-treated HaCaT keratinocytes. Moreover, AGEs-BSA stimulation promotes the activation of ERK1/2 and RAS through mevalonate pathway, thereby demethylating the -562bp site of MMP-9 promoter and upregulating MMP-9 level. Interestingly, the HMG-CoA reductase inhibitor simvastatin blocks demethylation at the -562bp site (63). Furter studies illustrated that TET2, a DNA demethylation enzyme, is elevated in AGEs-BSA-stimulated human primary keratinocytes, while the methylation of the MMP-9 promoter is decreased. TET2 can directly bind to a segment around the transcriptional start site in the MMP-9 promoter domain and regulate its expression, thus affecting the migration and proliferation of skin keratinocytes (64). Therefore, it suggests that the MMP-9 promoter DNA demethylation would pass though the mevalonate pathway to TET-2. Interestingly, another study proved that GADD45a plays an important role in demethylation of the MMP-9 promoter, which is augmented in diabetic wound and AGEs-induced HaCaT cell. Nevertheless, GADD45a knockout suppresses AGEs-induced increase of MMP-9 and demethylation of the MMP-9 promoter, and enhances the HaCaT cells migration without influencing the apoptosis and proliferation of HaCaT cells, whereas overexpression of GADD45a improves the MMP-9 promoter demethylation. Remarkably, HG induces the binding of GADD45a to MMP-9 promoter and promotes GADD45a to thymine-DNA glycosylase recruitment for base excision repair-mediated demethylation (65). Altogether, these findings reveal that targeting the site-specific demethylation of MMP-9 promoter through interfering with mevalonate pathway, ERK1/2 and RAS signals, TET-2, as well as GADD45a is a promising therapeutic strategy to modulate the aberrant expression of MMP-9 during diabetic ulcer.

### 2.9 Endogenous substance and MMPs

Substance P (SP) is a type of neuropeptide consisting of 10-amino acid, which has been demonstrated to accelerate the skin wound repair under diabetic condition. Compared with non-diabetic acute wounds, impaired re-epithelization, decreased formation of granulation tissue, and suppressed re-vascularization can be observed in diabetic wounds in mice, accompanied by the reduction of SP, up-regulation of MMP-9, as well as elevation of cytokines associated with inflammation in wound fluids (66). SP facilities the wound healing in DM rats through relieving inflammation, promoting fibroblast proliferation, promoting COL deposition and improving angiogenesis, which up-regulates the expressions of IL-10 and HO-1, down-regulates TNF-α, IL-1β, as well as MMP-9, and enhances neovascularization *via* elevating VEGF, TGF-β1, and eNOS (67). In short, these findings indicate that SP may possess great potential in treating diabetic cutaneous wounds.

Leucine-rich α-2-glycoprotein-1 (LRG1), as a crucial factor that participates in angiogenesis as well as cutaneous wound repair, has been proved to be down-regulated in the corneal epithelium of DM mice and in HG-treated TKE2 cells. Nevertheless, exogenous administration of LRG1 improves corneal re-epithelialization, nerve regeneration, and wound healing through up-regulating MMP-3 and MMP-13, which is accompanied by the activation of JAK2/STAT3, AKT, EGFR as well as TGF-β3 signaling. Remarkably, these protective effects of LRG1 are abrogated by MMP-3 and MMP-13 inhibitors, indicating that LRG1 accelerates wound repair in diabetic corneal epithelium *via* modulating MMPs (68). Thus, targeting LRG1/MMPs axis is a promising strategy for wound healing in diabetic keratopathy.

Angiotensin II is a type of fibrogenic factor that modulates COL metabolism and capillary formation of skin cells by regulating Ang II type 1 (AT1) and AT2 receptors. Ren et al. reported that Angiotensin II up-regulates the expression levels of TIMP-1, TGF-β, COL I, and COL III in diabetic skin fibroblasts without influencing the expression of MMP-1, thus leading to the imbalance of MMP-1 and TIMP-1, as well as the improvement of COL synthesis. However, losartan, an AT1 receptor blocker, suppresses the effects of Angiotensin II (69), which demonstrates that targeting Angiotensin II/AT1 receptor and TGF-β-associated axis may contribute to the balance of MMP-1 and TIMP-1 in diabetic skin.

Cytochrome P450 (CYP) epoxygenases play a critical part in diabetic wound healing, which catalyzes arachidonic acid to produce epoxyeicosatrienoic acids (EETs). Zhao et al. found that CYP2C65 and CYP2J6 are obviously decreased in the granulation tissues in *ob/ob* mice, leading to the down-regulation of EETs, the aggravation of inflammation, and the inhibition of angiogenesis. However, exogenous EETs administration down-regulates the levels of TNF-α, IL-6, as well as IL-1β, the expressions of MMP-9, and the infiltration of neutrophil and macrophage, resulting in the improvement of wound healing, angiogenesis and COL deposition (70). Therefore, these findings reveal that targeting CYP epoxygenases-mediated MMP-9 expression is a potential option for diabetic wound healing.

Aldose reductase is the first enzyme present in the polyol pathway, whose inhibition plays a paramount part in the progression of diabetic keratopathy in humans. Aldose reductase inhibitor treatment facilitates the corneal wound healing in galactose-induced diabetic rats by inhibiting sorbitol accumulation, down-regulating the gene and protein expressions of MMP-10, and up-regulating the protein expression of integrin α3. Notably, topical treatment with the recombinant MMP-10 impairs the wound healing in DM rats (71), which reveal that targeting MMP-10 is a promising option to improve wound healing in diabetic retinopathy.

In addition to TIMPs, neutrophil gelatinase-associated lipocalin (NGAL) is another significant regulator of MMP-9, which forms a complex with MMP-9, stabilizing it and preventing its degradation. NGAL/MMP-9 complex attenuates the diabetic wound healing in DM rats by facilitating the inflammation *via* up-regulating MMP-9 expression. Abdollahi et al. observed that the wound healing rate is decreased, whereas the number of neutrophils in tissue and circulating, as well as the expression of NGAL, MMP-8, as well as MMP-9 are elevated in diabetic group. However, insulin reverses these phenomena induced by HG. Besides, insulin also down-regulates the pro-inflammation factors TLR4, TLR2 and TNF-α in diabetic skin wound granulation tissue (72). Thus, these findings suggest that targeting NGAL/MMP-9 complex may be a potential therapy for diabetic wound repair.

## 3 Clinical research studies of MMPs in regulating diabetic ulcer

High levels of MMP-9 in serum, wound fluid, as well as skin tissue of diabetic individuals is a signal that may indicate the poor healing process and connected with failed dermal grafting of DFUs (73). Jindatanmanusan et al. reported that MMP-9 contents in wound fluid from poor healers are dynamic and obviously higher than those of good healers, while the MMP-9 remained at a lower level throughout the treatment period in the good healer group (74). Notably, the original MMP-9 level at week 0 proved to be a predictor of good/poor healing during the 12-week follow-up. Besides, compared with patients with non-healing DFUs, the levels of pro-MMP-9 and active-MMP-9 in wound fluid of patients with healing DFUs were significantly reduced, while TIMP-1 and TGF-β1 were significantly increased (75). These results suggest that the elevated MMP-9/TIMP-1 ratio affects the healing of DFUs. Interestingly, single nucleotide polymorphism (SNP -1562C>T) (rs3918242) in the promoter region of MMP-9 gene that modifies the transcriptional activity of MMP-9 is relevant to the development of DFUs. Singh et al. reported that Increased frequency and expression of T allele of SNP -1562C>T in MMP-9 gene are related to up-regulation of MMP-9 in wound fluids of T2DM patients, which results in degradation of matrix and the development of chronic wound (76). Trøstrup et al. found that there are no significant differences between MMP-9 levels in the wound fluid from patients with venous leg ulcers (VLUs) and patients with DFUs, but are both higher than that of healing wounds. Notably, serum levels of MMP-9 in patients with DFUs are higher than that of patients with VLUs (77).

Furthermore, increased ratio of serum MMP-9/TIMP-1 has been proved to predict poor wound healing in DFUs. The level of MMP-9 in the serum of good healers is lower than poor healers at first visit, and it reduces about 5-fold after 4-week therapy, while the serum level of MMP-9 in the poor healer shows little change. Remarkably, the MMP-9/TIMP-1 ratio can better reflect the healing before therapy and after 4-week therapy compared with MMP-9 (78). Dinh et al. reported that higher levels of TNF-α, monocyte chemoattractant protein-1 (MCP-1), MMP-9, as well as fibroblast growth factor (FGF)-2 can be detected in the serum of individuals whose ulcers are unable to heal. In addition, the results of skin biopsy analysis demonstrated that diabetic individuals have elevated immune cell infiltration, as well as increased MMP-9 expression, which adversely modulates the signals related to insulin, leptin, as well as growth factors (79). Moreover, MMP-9 is up-regulated in the skin tissue of diabetic wounds with bacterial infection compared with nondiabetic patients with wounds, while that of TIMP-1 as well as VEGF is down-regulated, which indicate that an excessively high ratio of MMP-9/TIMP-1 contributes to delayed wound healing in infected DFUs through reducing VEGF levels (80). The activity of MMP-9 as well as A Disintegrin and A MetalloProtease Domain 17 (ADAM17)/TNF-Alpha Converting Enzyme (TACE) is proved to be enhanced in ischemic diabetic wound biopsies compared with neuropathic biopsies, while the mRNA levels of MMP-9 and ADAM17/TACE are comparable between the two groups. Importantly, TIMP-3 is significantly lower in ischemic samples, which indicates that increased protein hydrolysis milieu may be a trigger for diabetic ulcer development (81).

**Figure 2:**
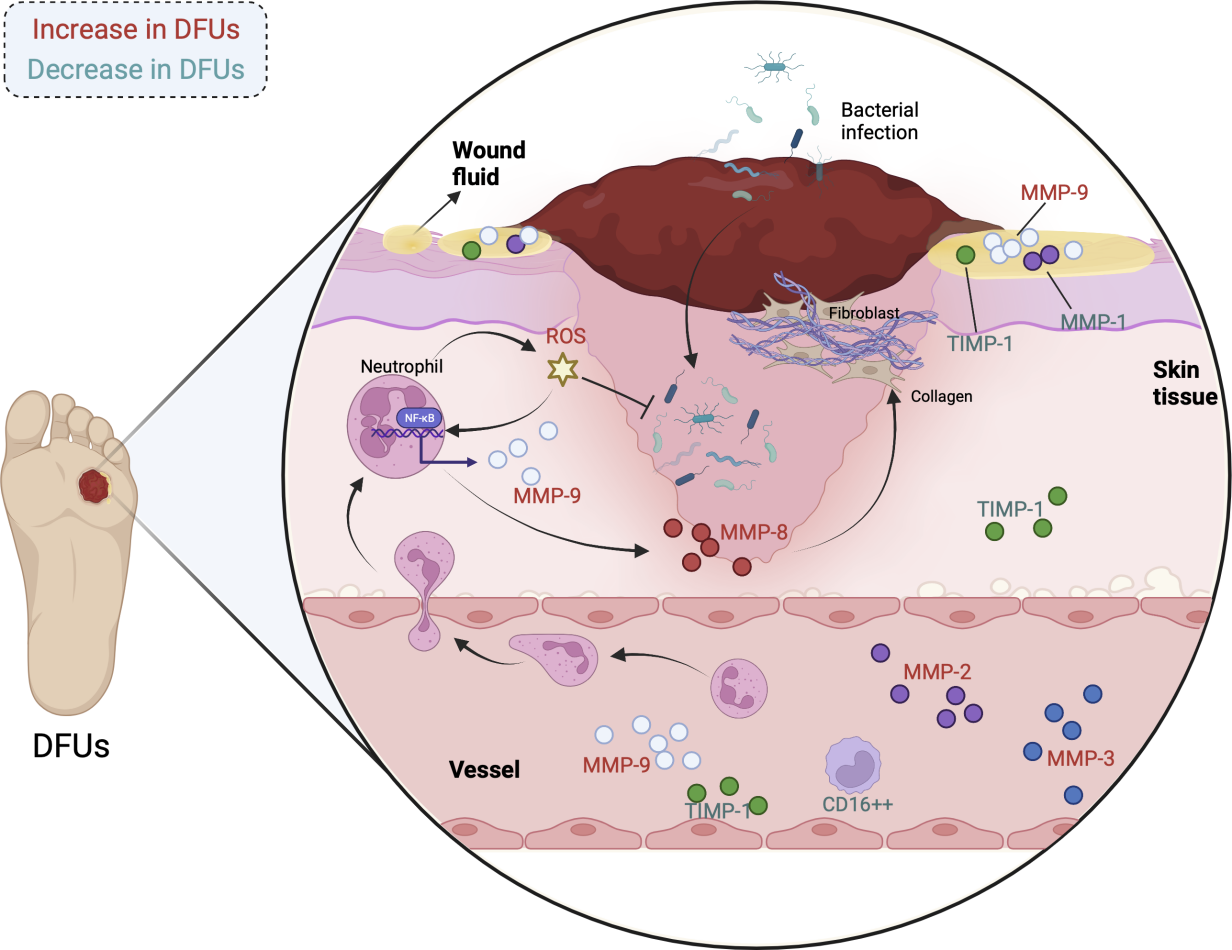
The changes of MMPs levels in individuals with diabetic ulcers. (MMPs are influential regulators in DFUs, whose expressions are diverse in the wound fluid, skin tissue, and blood of patients. The correlations have been observed between MMPs and physiological as well as pathological processes of DFUs, involving COL deposition, inflammatory response, oxidative stress, and bacterial infection. The MMPs in red are increased in individuals with DFUs, while the MMPs in green are decreased in individuals with DFUs.).

In addition to MMP-9, MMPs contains MMP-1, MMP-2, MMP-3, as well as MMP-8 are illustrated to play indispensable roles in individuals with diabetic ulcer. The expressions of MMP-1, MMP-9, and TIMP-1 in diabetic individuals are in dynamic change during the wound healing process. The initial levels of MMP-1 as well as the MMP-1/TIMP-1 ratio are obviously higher in wound fluid from DFU patients with better wound healing, while that of MMP-9 is significantly lower in these patients. Besides, the MMP-1 level starts to elevate at week 4 in patients with better wound healing and is followed by a reduction at week 8, whereas that of MMP-1 is stable in patients with poor wound healing (82). Thus, MMP-9, MMP-1, as well as TIMP-1 may be useful biomarkers for DFU therapy at the first patient visit. Min et al. reported that faster wound closure rate is relevant to lower plasma MMP-2 and MMP-9 at week-4 and week-8 visits. In addition, the percentage of CD16^++^ monocytes is negatively correlate with plasma MMP-2 and pro-MMP-9, but is positively related to the percentage of CD163 monocytes. Remarkably, MMP-9 and percentage of CCR2^+^ are significantly decreased, while non-classical percentage of CD16^++^ and MMP-3 are increased in the DFUs healing group after 8 weeks compared with the DFUs non-healing group (83). Therefore, MMPs and non-classical percentage of CD16^++^ may be biomarkers for detecting the degree of healing of DFUs. Besides, Kupczyk et al. found that the serum levels of MMP-2 and MMP-3 in diabetic individuals with ulcer are obviously higher than those in the control group, which may serve for the delayed healing of chronic wounds and the aggravation of vascular complications (84). Another study illustrated that MMP-9 levels in the wounds of diabetic individuals are parallel to NF-κB p65. When skin injury occurs, a mass of neutrophils will be mobilized to the site of injury, which release cytokines like MMP-8, MMP-9, and ROS to resist bacterial infection as well as modulate thrombus formation. Interestingly, excessive ROS activates NF-κB signal, which subsequently triggers the up-regulation of MMP-9 and eventually the delayed wound healing. On the contrary, MMP-8 contributes to the COL deposition and ECM formation (85). The changes of MMPs levels in individuals with diabetic ulcers are shown in **Figure 2**.

## 4 Regulation of MMPs in diabetic ulcer by natural products

### 4.1 Flavonoids

**Figure 3:**
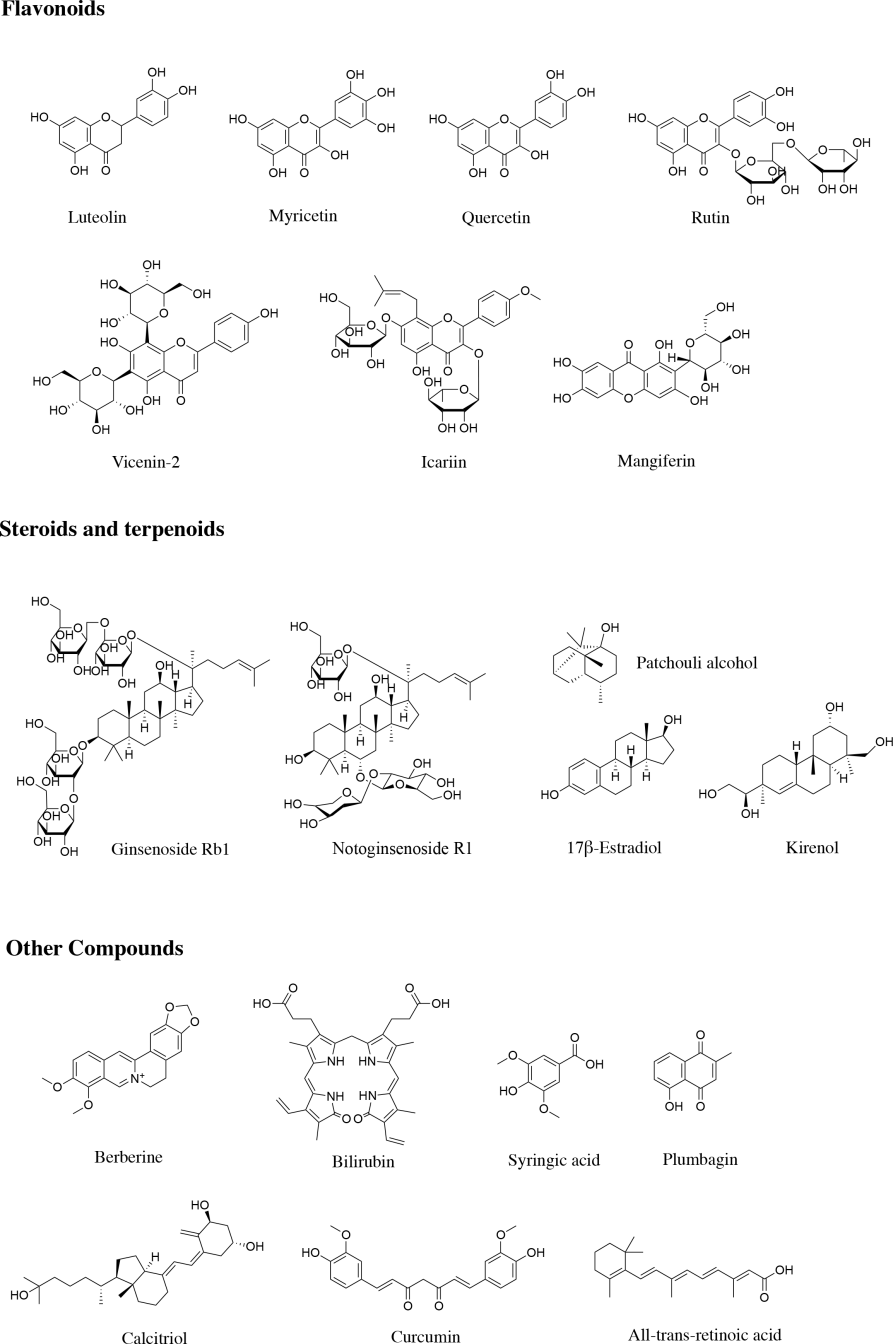
Structures of natural products regulating MMPs in diabetic wound healing.

Luteolin (**Figure 3**) is a critical flavonoid extracted from numerous of plants, such as leaves of M. annua Linn., which is reported to reduce blood glucose levels, enhance cutaneous wound healing process, and accelerate skin wounds re-epithelization in diabetic rats through attenuating inflammation and oxidative stress. Luteolin represses the infiltration of inflammatory cell, reduces the levels of TNF-α, IL-6, IL1-β and MMP-9 *via* down-regulating the NF-κB signaling pathway. Meanwhile, luteolin brings down the expressions of SOD1 and glutathione peroxidase (GSH-Px), as well as p-Nrf2 to modulate oxidative stress (86). Therefore, these phenomena indicate that luteolin may be a possible agent to treat diabetic wound injury by targeting NF-κB/MMP-9 axis and Nrf2-meidated anti-oxidant system.

Myricetin (**Figure 3**), a bioflavonoid widely presents in a variety of plants, tea, fruits as well as vegetables, is demonstrated to exert numerous biological activities, especially preventing cellular oxidative stress through regulating antioxidant enzymes. Recent study found that myricetin intervention obviously mitigates the damages on dermal fibroblasts stimulated by DM through modulating MMPs. Myricetin inhibits the formation of MMP-1, MMP-2, and MMP-9 in diabetic fibroblasts, and suppresses catalase (CAT) and SOD. In contrast, myricetin increases the mRNA expression of TIMP-1, resulting in the increase of the ratio of TIMP1/MMPs in diabetic fibroblasts. Additionally, pro-COL I and III levels in diabetic fibroblasts are enhanced after myricetin intervention, which is conducive to diabetic wound repair (26). All in all, myricetin can relive DM-induced damages on dermal fibroblasts, which implies that myricetin may function as a drug candidate to accelerate wound healing under in patients with DM.

Quercetin (**Figure 3**) is a naturally occurring flavonoid compound with a variety of bioactivities, covering anti-ulcer, anti-inflammation, and cardiovascular protection. Recently, several studies reveal that quercetin displays promising effect on diabetic ulcer (87), which accelerates the wounds closure and reduces wound diameter through down-regulating the pro-inflammatory cytokines and enzymes, including TNF-α, IL-1β, as well as MMP-9, while up-regulating IL-10, VEGF, and TGF-β. In addition, topical application of quercetin improves the formation of thick granulation tissue with more new blood vessels, accelerates re-epithelialization and fibroblast conversion into the phenotypic of myofibroblast, promotes the COL synthesis, and deposition and orientation in wounds of diabetic rats (88). Thus, its suggests that quercetin exhibits great potential to mitigate diabetic ulcer.

Rutin (quercetin-3-O-rutoside, **Figure 3**) is a common flavonoid that can be found in the seeds, stems, leaves, as well as flowers of buckwheat, which improves wound healing in hyperglycemic rats *via* preventing oxidative stress and inflammatory response. Specifically, rutin intervention obviously relieves the body weight loss and metabolic dysfunctions in rats induced by DM, decreases the number of inflammatory cells, promotes the activity of Nrf-2 as well as the expression of related antioxidant enzymes such as SOD1 and GSH-Px, down-regulates the levels of TGF-β1, MMP-2, MMP-9, NF-κB, IL-1β, IL-6, TNF-α, and VEGF, and elevates the expression of neurogenic-related protein (89). Thus, these results reveal that targeting NF-κB-mediated MMPs axis is one of the crucial mechanisms of rutin in regulating diabetic wound healing, and rutin could function as a potential drug for DFUs.

Vicenin-2 (VCN-2, **Figure 3**) is a flavonoid glycoside separated from numerous natural plants, which attenuates oxidant stress and inflammatory, and improves epithelialization as well as cell remodeling. More recently, studies illustrated that VCN-2 intervention facilities wound healing in STZ-induced DM rats through improving cells proliferation as well as reducing the inflammatory cells, which down-regulates the expressions of pro-inflammatory cytokines *via* NF-κB signal pathway. Meanwhile, VCN-2 augments the number of fibroblast cells and neoangiogenesis *via* down-regulating the levels of MMP-9 and anti-HIF-1α *via* VEGF and TGF-1β signal pathway. Compared with diabetic group, VCN-2 treatment decreases the levels of blood glucose, reduces food and fluid intakes, while increases insulin levels, body weight, as well as the percentage of wound closure (90). Altogether, it suggests that VCN-2 may be an available agent for diabetic wound repair *via* modulating NF-κB, VEGF, and TGF-1β signal pathways.

Icariin (**Figure 3**) is a pivotal flavonoid derived from *Epimedium* genus with various bioactivities, including anti-cancer, anti-inflammation, and pro-angiogenesis. Icariin administration improves wound repair in diabetic rats through down-regulating the protein expressions of NF-κB, TNF-α, MMP-2, as well as MMP-9, elevating the levels of IL-10, up-regulating the expression of CD31, and increasing the relative COL deposition in the healing tissue. In short, icariin contributes to the progression of diabetic wound healing *via* alleviating inflammation, improving angiogenesis, and promoting normal ECM formation as well as remodeling in the healing tissue (27), which suggest that icariin may serve as a promising agent for diabetic ulcer.

Mangiferin (**Figure 3**) a well-known naturally occurring polyphenol widely distributed in various plant species, is demonstrated to exert numerous bioactivities, particularly preventing cancer and DM. Lwin et al. reported that mangiferin application attenuates the ROS-induced oxidative stress, lessens the wound area, and increases the skin thickness of around the wound. In addition, mangiferin elevates EGF, FGF, TGF-β, VEGF, PI3K, and Nrf-2 protein expression in diabetic wound, while reduces the expression of MMP-2, TNF-α and NF-κB p65, suggesting that mangiferin can shorten the inflammatory phase of wound tissue under hyperglycemia (91). Therefore, mangiferin is a potential agent for promoting wound repair in individuals with DM, and targeting MMP-2 is an underlying mechanism of mangiferin in treating diabetic ulcer.

### 4.2 Steroids and terpenoids

Ginsenoside Rb1 (G-Rb1, **Figure 3**), an active substance widely existed in *Panax ginseng*, has been demonstrated to possesses numerous pharmacological activities, including anti-inflammation, antioxidant, and antimicrobial effects. Recent studies revealed that G-Rb1 displays promising effect on diabetic chronic wounds healing *in vitro*, which significantly increases cell proliferation and COL synthesis. Specifically, G-Rb1 up-regulates VEGF, TGF-β1, TIMP-1 in cultured fibroblasts from patients with DFUs. Interestingly, TGF-β1 and TIMP-1 may increase COL synthesis, and VEGF improves the formation of thick granulation tissue with more new blood vessels in G-Rb1-treated diabetic (92). Taken together, it suggests that G-Rb1 is a candidate agent for the wound-healing activity of diabetic fibroblasts. Nevertheless, further *in vivo* studies are required to investigate the activity of G-Rb1 on diabetic would healing.

Notoginsenoside R1 (NR1, **Figure 3**), a dominating bioactive ingredient separated from Panax notoginseng, is demonstrated to facilitate wound healing in diabetic rats by obviously accelerating the wound closure rate, increasing ECM secretion, elevating COL growth, up-regulating the expression of CD31, and down-regulating the expression of cleaved caspase-3. More importantly, NR1 administration gives rise to the down-regulation of MMP-9, MMP-3, IL-1β, and IL-6, while up-regulation of TIMP1 as well as TGF-β1. The results of RNA-Seq technology illustrate that NR1 mainly influence ECM related processes and inflammation in diabetic wound healing by targeting TIMP-1 and MMP-3 (93). Thus, these phenomena indicate that NR1 may be a feasible candidate agent for diabetic ulcer *via* regulating MMP-mediated signaling pathway.

Patchouli alcohol (PA, **Figure 3**) is a bioactive ingredient separated from patchouli, with anti-inflammatory and anti-influenza effects. Recently, several studies reveal that PA administration improves wound healing in HFD-fed mice by up-regulating TGF-β1, MMP-2, MMP-9, COL1A1, down-regulating the levels of NF-κB, p-IκB, TNF-α as well as MCP-1, promoting adenosine monophosphate activated protein kinase (AMPK) phosphorylation and relieving inflammation. Simultaneously, PA intervention significantly reverses the decreased viability of LPS-induced HaCaT cells, impaired cell migration and proliferation, increases AMPK phosphorylation and activates TGF-β1 pathway in a dose-dependent manner. Notably, TGF-β1 siRNA blocks the effect of PA on LPS-induced HaCaT cells (94). Therefore, it suggests that PA is a candidate agent for obesity or insulin resistance, which facilitates diabetic wound healing through relieving inflammatory response.

17β-Estradiol (E2, **Figure 3**) is one of the most essential forms of estrogen, which is demonstrated to control the expressions of COL and MMP, and regulate cytokines, growth factors, as well as ECM turnover in diabetic wound healing. Recently, Pincus et al. investigates the regulatory effects of E2 in *db/db* mice, and they found that topical E2 treatment accelerates cutaneous wound healing by modulating the expressions of MMP. E2 can not only directly reduces the levels of MMP-13 and MMP-2, but also indirectly declines MMP-13 and MMP-2 *via* decreasing the MMP-14, pro-MMP-2. Besides, uterine weight and COL fibers deposition augmented in E2-treatment group compared with placebo treated mice. In addition, E2 treated up-regulates the expressions of estrogen receptor-α (ER-α) (95). Therefore, these findings illustrate that E2 may act as a therapeutic agent for diabetic wound injury by targeting ER-α-mediated signaling pathway.

Kirenol (**Figure 3**) is an important diterpenoid separated from the medicinal plant *Siegesbeckia orientalis*, which possesses various bioactivities, including anti-inflammation, anti-rheumatism, and wound healing. Kirenol administration reverses the up-regulation of angiogenesis-associated genes MMP-2 and MMP-9 in wounds of STZ-induced DM rats, decreases inflammation-related genes NF-κB, cyclooxygenase-2 (COX-2), as well as iNOS, reduces the contents of malonaldehyde (MDA), while increases the activities of antioxidant enzymes, which result in the alleviation of oxidative trauma. In addition, the results of histopathological examination demonstrate that kirenol intervention gives rise to the decline of inflammatory cell infiltration, elevation of fibroblasts, new blood vessels, as well as granulation tissue configuration (96). In short, these evidences illustrate that kirenol is promising for improving wound curing in diabetic ulcer.

### 4.3 Polysaccharide and glucoside

Hsian-tsao polysaccharides (WEP) are the major functional component in *Mesona procumbens* Hemsl., which has potent anti-oxidant and anti-inflammatory effects. Recently, several studies revealed that Hsian-tsao extracts (EE) and WEP displays promising effect on wound healing in diabetic, which decrease crust and improve the formation of thick granulation tissue with more new blood vessels, and re-epithelialization. Specifically, EE and WEP up-regulate IL-8, MIP-2, MCP-1, TIMP-1, as well as VEGF, down-regulate MMP-2 and MMP-9, and suppress MG-triggered protein glycation and ROS accumulation. Furthermore, both EE and WEP enhance the methylglyoxal (MG)-impeded phagocytosis of *Staphylococcus aureus* and *Pseudomonas aeruginosa* driven by macrophages, which maybe improve impaired wound healing. Interestingly, WEP is more effective on regulating the factors associated with diabetic wound repair than EE (97). Therefore, it suggests that EE and WEP are the candidate agent for chronic diabetic wounds.

*Dendrobium* Polysaccharides (PDC) is the main bioactive substance of Dendrobium candidum, which has anti-tumor and anti-aging effects. Recently, these studies revealed that PDC at the concentrations of 100, 200, as well as 400 μg/mL elevates the level of COL, improves the viability of human skin fibroblasts, suppresses the cell apoptosis induced by HG, accompanied by the elevation of TIMP-2 and reduction of MMP-2. Therefore, these results confirmed that PDC can display protective effects on diabetic ulcer *in vitro*, and the mechanism may be related to the modulation of TIMP-2 and MMP-2, which provides a new idea for the prevention and treatment of diabetic skin ulcer or wound (98). However, further *in vivo* researches are required to clarify the activity of PDC against diabetic wound healing.

### 4.4 Other compounds

Berberine (**Figure 3**) is a naturally occurring alkaloids separated from *Coptis chinensis* Franch. and has been demonstrated to possesses various pharmacological activities, including anti-microorganisms, anti-obesity, and improving insulin resistance (99). Recent studies found that berberine displays promising therapeutic effect on diabetic ulcer, which alleviates HG-induced HaCaT cell damage and enhances cell proliferation by activating thioredoxin reductase 1 (TrxR1)/c-Jun N-terminal kinase (JNK) pathway, and relieves oxidative stress and apoptosis through increasing GSH, SOD, and total antioxidant capacity (T-AOC), while down-regulating ROS, MDA, TUNEL-positive rate as well as caspase-3 activity. Notably, topical berberine application promotes the wound healing and elevates ECM synthesis in T2DM rats stimulated by HFD and STZ *via* decreasing MMP-9 and elevating TGF-β1 and TIMP-1 (28). Therefore, these findings illustrate that the wound healing effect of berberine against diabetic ulcer might be conferred through modulating TrxR1/MMP9 signaling pathway.

Bilirubin (**Figure 3**) is final metabolite of heme in mammals, which promotes wound healing by ameliorating oxidant stress, inflammation and angiogenesis. Recent study illustrated that bilirubin accelerates wound repair by facilitating COL fibers deposition, granulation tissue formation and contraction, induce neoangiogenesis and anti-inflammatory in STZ-induced diabetic rats. Bilirubin intervention alleviates inflammation through augmenting the expressions of IL-10 and decreasing IL-1β, which improves the angiogenesis and wound closure *via* up-regulating the levels of TGF-β1, HIF-1α, VEGF, IL-10 and SDF-1α and down-regulating TNF-α and MMP-9. Meanwhile, the results of histopathological assay indicate that re-epithelization of skin wound in bilirubin-treated group better than the control group (100). Thus, these results display bilirubin enhances skin wound healing in DM rats through balancing the levels of factors-associated with the process of wound closure.

Syringic acid (**Figure 3**), a critical phenolic compound synthesized *via* shikimic acid pathway in plants, is widely distributed in numerous edible plants like olives, pumpkin, and grapes. Syringic acid administration accelerates the wound closure rate and epithelization of diabetic wounds in rats, accompanied by the increase of hydroxyproline content and total protein levels. In addition, 14 days after syringic acid intervention, the inflammation and oxidative stress in diabetic wounds are alleviated, as evidenced by down-regulation of p65, IL-8, TNF-α, IL-2, IL-1β, MDA, and elevation of IL-10, Nrf-2, Keap1, as well as antioxidant enzyme activities. Intriguingly, syringic acid significantly reduces MMP-2, MMP-8, and MMP-9, up-regulates TIMP-1 and TIMP-2, elevates the contents of TGF-β1, COL I, α-SMA, CD31, CD68, as well as VEGF in diabetic wounds (29). Thus, its suggests that syringic acid exhibits great potential to mitigate diabetic ulcer.

Plumbagin (**Figure 3**), one of the bioactive constituents separated from the roots of *Plumbago zeylanica*, has emerged as a promising agent for diabetic wound healing. Plumbagin administration significantly promotes the wound closure as well as contraction of diabetic rats through accelerating epithelialization and the deposition of COL, promoting the secretion of insulin, improving the antioxidant status,and lowering lipid peroxides and lipid levels while elevating the HDL level Specifically plumbagin up-regulates the expression of Nrf-2, COL I, TGF-β as well as a-SMA down-regulates the expression of Keap1and rescues the decreased activities of the antioxidant enzymes in diabetic rats. Interestingly, plumbagin also increases EGF, VEGF and FGF, decreases MMP-2, COX-2, iNOS, CD8, CD163, as well as NF-κB p65, and suppresses IL-6 and IL-1β (101). Thus, alleviating inflammation and oxidation-induced injury is a possible mechanism of plumbagin in diabetic ulcer therapy.

Calcitriol (**Figure 3**) is the active form of vitamin D, which regulates the proliferation and differentiation of keratinocytes. Recent studies demonstrate that Calcitriol exerts promising protective effect on diabetic wound healing by targeting MMPs. The slower wound healing during DM is in connection with the up-regulation of MMP-1, MMP-9, and TIMP-1, as well as the down-regulation of MMP-8 and MMP-10 in wound tissue. Calcitriol intervention leads to the decrease of MMP-1 and MMP-10 levels, and contributes to wound healing in primary keratinocytes from the patients with DFUs (102). Therefore, Calcitriol may serve as a feasible modulator of MMP expression to accelerate wound healing in DM.

Curcumin (**Figure 3**) is a naturally occurring diketone compound principally extracted from the rhizomes of some plants in Zingiberaceae and Araceae, with potent anti-inflammatory and anti-cancer properties. Recently, several studies revealed that curcumin displays promising effect on diabetic ulcer, which accelerates the wounds closure through down-regulating the pro-inflammatory cytokines and enzymes, including TNF-α, IL-1β, and MMP-9, up-regulating IL-10 levels, and elevating the activities of SOD, CAT, as well as GSH-Px. Besides, topical administration of curcumin improves thick granulation tissue formation with more new blood vessels and fibroblasts, and promotes the COL synthesis, deposition and orientation in diabetic wounds (103). In addition, another study reported that combination of substance P (SP) and curcumin is a potential strategy for diabetic wound healing. SP is derived from the body and has the function of regulating angiogenic factors. Combination of SP and curcumin intervention promotes the formation of thick granulation tissue, reduces wound diameter, increases fibroblasts, and accelerates COL synthesis, deposition, as well as orientation in wounds of diabetic rats. In addition, combination of SP and curcumin facilitates new blood vessels formation through the levels of VEGF, TGF-β1, HIF-1α, SDF-1α, HO-1 as well as eNOS. Simultaneously, after combination of SP and curcumin administration, the inflammation and oxidative stress in diabetic wounds are alleviated, as evidenced by the down-regulation of TNF-α, IL-1β and MMP-9, and the elevation of IL-10, SOD, GSH-Px, growth associated protein-43 (GAP-43) and CAT activities (104). Therefore, it suggests that curcumin is a candidate agent for diabetic ulcer, which promotes wound healing through relieving inflammatory response and oxidative stress.

All-*trans*-retinoic acid (RA, **Figure 3**), an intermediate product of vitamin A metabolism in animals, exerts a broad spectrum of bioactivities. RA can moderate the skin of chronological aging process by declining the levels of COL-degrading MMPs and augmenting the COL. Interestingly, RA treatment was established that accelerate the diabetic wound healing in organ culture through improving epidermal hyperplasia, elevating soluble COL and pro-COL production, as well as down-regulating the expressions of active MMP-9 and active MMP-1 and up-regulating TIMP-1. Meanwhile, Lateef et al. also reported the results of TIMP-1 inhibiting MMP function are similar to RA intervention. It is likely that RA attenuates the function of MMP *via* increasing the level of TIMP-1 (105). Taken together, although these findings indicate RA possibly be an agent to treat diabetic wound injury, further experiments are needed to clarify the specific mechanisms.

Relaxin, a peptide hormone with the molecular weight of 6 kDa, can improve wound healing under diabetic condition. Relaxin intervention elevates the mRNA and protein contents of VEGF in wounds from diabetic mice on postoperative day 3 and 6. Daily treatment of relaxin improves the levels of SDF1-α, accelerates healing process in the wounds of diabetic mice, shortens the time of complete wound closure through mediating VEGF and SDF1-α. Further studies demonstrated that treatment of relaxin markedly increases the level of microvessel density, augment levels of VEGFR-2, vascular endothelial cadherin, MMP-11, and enhances immunostaining of CD34 and VEGFR-1 in both non-diabetic and diabetic mice. More importantly, the results from clinical observation displayed that relaxin administration represents an alternative therapeutic regimen without any side effects (106). Thus, relaxin may possess a promising application in diabetic wound healing.

Exendin-4 is a polypeptide hormone isolated from the saliva of the *Heloderma suspectum*, which is proved to improve the transcription level of insulin gene, stimulate the release of insulin, and control blood glucose concentration. Recently, Exendin-4 was reported to possesses potent wound healing activity in DM, which facilities the wound healing in spontaneously diabetic ZDF rats *via* attenuating inflammation, promoting fibroblast/myofibroblast activities, and augmenting total COL content *via* decreasing the CRP concentration and the level of MMP-9, as well as elevating the level of TIMP-1. However, exendin-4 at the concentration of 100 nM suppresses fibroblast/myofibroblast metabolic activity and reduces COL production, which suggests that high exendin-4 doses display a cytotoxic effect (107). Another study showed that Glucagon-like peptide (Glp)-1 analogue exendin (Ex)-4 improves chronic gastric ulcer through suppressing inflammation and promoting angiogenesis in STZ-induced diabetic rats, which ameliorates the polymorphonuclear leukocytes (PMN) infiltration, up-regulates the levels of MCP-1, IL-10, eNOS, and cAMP, while down-regulates the levels of MMP-2, myeloperoxidase, superoxide anions, and IL-1β (108). Thus, these results demonstrate that exendin-4 is a potential therapeutic option for diabetic wound repair, but the safety doses need further investigation.

## 5 Conclusions and future directions

**Figure 4:**
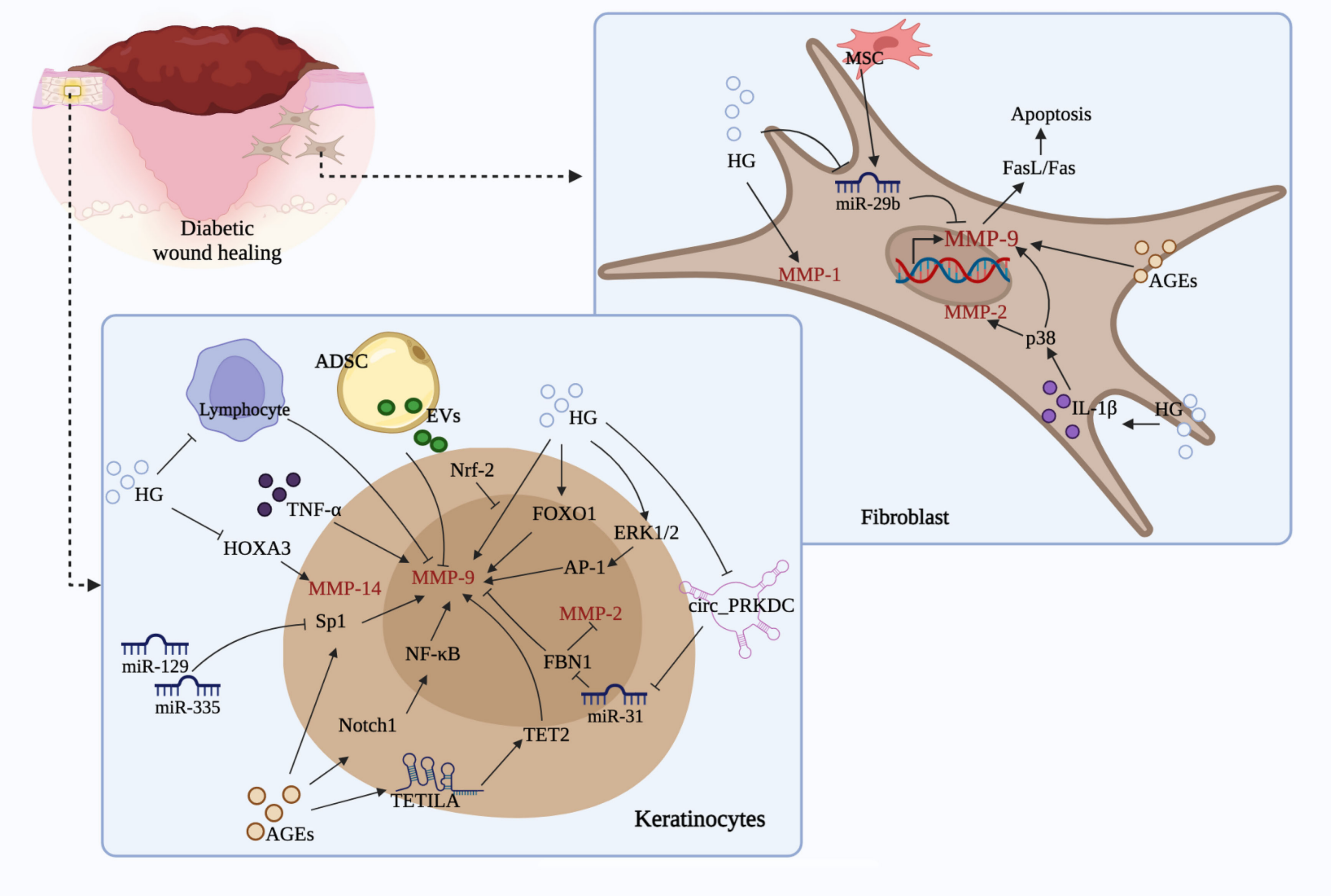
Regulation of MMPs by different cell types and related mechanisms. (Stimulated by HG and AGEs, keratinocytes and fibroblasts could produce a large amount of MMP-9, MMP-2, MMP-1 and MMP-14, which leads to decreased activity and migration, and ultimately causes impaired wound healing. During this process, signals such as miRNAs, lncRNAs, circRNAs, ERK1/2, FOXO1, HOXA3, Sp1, Notch, NF-κB, p38, FasL/Fas, and cells like ADSC, MSC, as well as lymphocyte are involved, which may promote or slow down the production of the above mentioned MMPs and thus affect diabetic wound healing.).

MMP family plays an indispensable role in numerous biological processes, involving tissue remodeling and growth, wound repair, tissue defense mechanisms, as well as immune responses. Under diabetic condition, tissues are trapped in inflammatory phase; continuous intensive stimulation of inflammatory cytokines leads to the dysregulation of MMPs, which subsequently degrades growth factors and matrix proteins necessary for wound repair, resulting in delayed wound healing. Notably, the expression of MMPs in diabetic ulcers is influenced by various internal and external factors, including DNA methylation, miRNAs, lncRNAs, AGEs, TIMPs, SP, LRG1, CYP, NGAL, etc. Additionally, signaling pathways such as Notch1/NF-κB, ERK1/2, p38, CXCL16-CXCR6, NRf-2, uPA/uPAR, FOXO1, as well as FasL/Fas are demonstrated to be concerned with the expression of MMPs during diabetic wound healing, which mainly improve the processes associated with inflammation, oxidative stress, apoptosis, angiogenesis, ECM formation, and re-epithelization. Furthermore, stem cells-mediated MMPs expression is another significant mechanism of diabetic wound healing (**Figure 4**). In short, these evidences indicate that developing agents targeting MMPs and the related signals or pathways has important implications for diabetic ulcers therapy.

Natural products are essential modifiable factors that affect human health and disease. In the last decade, more and more researchers have focused on bioactive natural ingredients to address the emergency situation of diabetic ulcers. Natural products exhibit a lot of beneficial effects in diabetic ulcers via targeting MMPs, such as alleviating inflammatory infiltration and oxidative stress of the wound, promoting angiogenesis, as well as enhancing granulation tissue configuration, ECM secretion, and COL growth (**Table 1**). Interestingly, natural products have been regarded as outstanding regulators that target miRNAs and DNA methylation in DM and related complications including diabetic ulcer. However, whether natural products mediate the expression of MMPs by regulating DNA methylation or non-coding RNAs and thus facilitate the diabetic wound repair process needs further investigation. In addition, these compounds possess different structures, but some of them are able to regulate the same MMPs or signaling pathways, suggesting that studying the structure-activity relationships of these compounds is important for elucidating the potential mechanisms by which they regulate MMPs to act as anti-diabetic ulcer agents. More importantly, not all natural products mentioned above are free from toxicity or side effects, a small percentage of them, such as bilirubin, calcitriol, and all-*trans*-retinoic acid have been proved to cause damage to brain and liver or produce teratogenic effects in overdose (109–111). Thus, it suggests that the long-term toxicity of natural products needs further consideration due to the long healing time of diabetic wounds requiring prolonged administration.

**Table 1:** Natural products in diabetic wound healing targeting MMPs.

Compound	Dosage	Administration route	Model/Cells	Targets	Ref
Luteolin	100 mg/kg (14 days)	Intraperitoneal.	STZ-induced diabetic rats with incision in the skin on the dorsal thorax	TNF-α↓, IL-6↓, IL1-β↓, **MMP-9↓**, NF-κB↓, SOD1↓, GSH-Px↓, Nrf2↓, VEGF↓, UCH-L1↑	(86)
Myricetin	3 μM	–	Fibroblasts from female T2DM patient	**MMP-9↓, MMP-2↓, MMP-1↓**, TIMP1↑,	(26)
Quercetin	0.3% quercetin ointment (21 days)	Topically.	STZ-induced diabetic rats with incision on the back	TNF-α↓, IL-1b↓, **MMP-9**↓, IL-10↑, VEGF↑, TGF-β1↑	(88)
Rutin	100 mg/kg	Intraperitoneal.	STZ-induced diabetic rats with incision on the back	Nrf2↑, SOD1↑, GPx↑, TGF-β1↓, **MMP-2↓**, **MMP-9↓**, NF-κB↓, IL-1β↓, IL-6↓, TNF-α↓, VEGF↓	(89)
Vicenin-2	12.5, 25, and 50 μM (14 days)	Topically.	STZ-induced diabetic rats with incision on the dorsal	IL-1β↓, IL-6↓, TNF-α↓, VEGF↑, TGF-1β↑, NO↓, iNOS↓, COX-2↓, NF-κB↓, **MMP-9↓**, anti-HIF1α↓	(90)
Icariin	0.04, 0.2, 1, and 5 ng/μg (14 days)	Topically.	STZ-induced diabetic rats with excisional wound on the back	IL-10↑, NF-κB↓, TNF-α↓, **MMP-2↓**, **MMP-9↓**	(27)
Mangiferin	1 and 2%	Topically.	STZ-induced type-2 diabetic male rat	EGF↑, FGF↑, TGF-β↑, PI3K↑, VEGF↑, TNF-α↓, Nrf2↑, **MMP-2**↓, NF-κBp65↓	(91)
Ginsenoside Rb1	10 ng/mL	–	Diabetic fibroblasts	VEGF↑, TGF-β1↑, TIMP-1↑	(92)
Notoginsenoside R1	0.038 mg/cm^2^ (15 days)	Topically.	HFD/STZ-induced diabetes rats with incision on the dorsum	Caspase-3↓, ECM↑, CXCL1↑, FOS↑, TGF-β1↑, **MMP-9↓**, IL-1β↓, IL-6↓, **MMP3↓**, TIMP1↑	(93)
Patchouli alcohol	20 mg/kg	Intraperitoneal.	HFD-fed mice	TGFb1↑, **MMP-2↑**, **MMP-9↑**, COL1A1↑, p-AMPK↑, NF-κB↓, p-IκB↓, TNFα↓, MCP-1↓,	(94)
	0~30 mg/mL	–	LPS-induced HaCaT cells		
17β-Estradiol	50 mg Estrogel 0.06% (7days)	Topically.	Female *db/db* mice with full-thickness wounds on the back	**MMP-13↓, MMP-2↓, MMP-14↓**, ER-α ↑	(95)
Kirenol	15% and 30% (14 days)	Topically.	STZ-induced diabetic rats with incision on the backside	NF-κB↓, COX-2↓, iNOS↓, **MMP-2↓**, **MMP-9↓**,	(96)
Hsian-tsao polysaccharides	100 μL	Topically.	STZ-induced diabetic mice	IL-8↑, MIP-2↑, MCP-1↑, TIMP-1↑, VEGF↑, **MMP-2↓, MMP-9↓,** ROS↓	(97)
	0~200 μg/mL	–	MG-induced RAW 264.7 cells and 3T3-L1 fibroblasts		
Dendrobium Polysaccharides	100, 200, and 400 μg/mL	–	HG-induced HSF cells	TIMP-2↑, **MMP-2↓**	(98)
Berberine	0.06 mg/ml (12 days)	Topically.	HFD/STZ-induced diabetes rats with incision on the dorsum	GSH↑, SOD↑, T-AOC↑, ROS↓, MDA↓, caspase-3↓, **MMP-9↓**, TGF-β1↑, TIMP1↑, ECM↑	(28)
	1.5625, 3.125, and 6.25 μM	–	HG-induced in HaCaT cells		
Bilirubin	0.3% bilirubin ointment (twice/days)	Topically.	STZ-induced diabetic rats with incision on the dorsal thoracic region	HIF-1α↑, VEGF↑, SDF-1α↑, TGF-β1↑, IL-10↑, TNF-α↓, IL-1β↓, **MMP-9↓,** MVD↑	(100)
Syringic acid	2.5% and 5% (14 days)	Topically.	STZ-induced diabetic rats with incision on the dorsal midline	Nrf2↑, Keap 1↑, MDA↓, SOD↑, CAT↑, GPx↑, GST↑, GR↑, collagen-1↑, α-SMA ↑, TGF-β↑, NF-κB↓, p65↓, IL-1β↓, IL-8↓, **MMP-2↓**, **MMP-9↓**, TNF-α↓, TIMP-1↓, TIMP-2↓, **MMP-8↓**, VEGF↑, IL-2↓	(29)
Plumbagin	10% and 20%	Topically.	STZ-induced diabetic rats with full thickness wounds on the back	Nrf2↑, TGF-β↑, α-SMA↑, Keap1↓, SOD↑, CAT↑, GPx↑, GR↑, GST↑, EGF↑, VEGF↑, FGF↑, **MMP-2↓**, COX-2↓, iNOS↓, CD68↓, CD163↓, NF-κBp65↓, NF-α↓, IL-6↓, IL-1β↓	(101)
Calcitriol	0.001 μM	–	Primary epidermal keratinocyte from DFUs	**MMP-1**↓, **MMP-10**↓	(102)
Curcumin	400 μl of curcumin 0.15% (19 days)	Topically.	STZ-induced diabetic rats with full thickness excisional wounds	TNF-α↓, IL-1β↓, **MMP-9↓**, IL-10↑, VEGF↑, TGF-β1↑, HIF-1α↑, SDF-1α↑, HO-1↑, eNOS↑, SOD↑, GPx↑, GAP-43↑	(103, 104)
	400 μl of curcumin 0.3% (19 days)				
All-*trans*-retinoic acid	0.75 g/ml providing at 2- to 3-day intervals, incubating 9 days	–	2-mm punch biopsies were obtained from hip skin of 16 diabetic patients	active **MMP-9↓**, active **MMP-1↓**, TIMP-1↑	(105)
Relaxin	25 μg/d (12 days)	Subcutaneously.	*db/db* mice with incisional wound on the back	VEGF↑, SDF1-α↑, p-eNOS↑, VEGFR-1↑, VEGFR-2↑, VE-cadherin↑, **MMP-11↑**	(106)
Exendin-4	week1: 3 μg/kg; week2: 6 μg/kg; week3: 10 μg/kg	Intraperitoneal.	HFD-induced diabetic rats with subcutaneous implantation of foreign material	CRP concentrations↓, **MMP-9**↓, TIMP-1↑,	(107)
	0~100 nmol/l	–	Fibroblasts/myofibroblasts obtained from rat wounds *in vivo* experiment		
	0.5 μg/kg/d (7 days)	Intraperitoneal.	STZ-induced diabetic rats with Acetic acid-induced chronic peptic ulcer	IL-10↑, eNOS↑, peNOS↑, **MMP-2**↓, cAMP↑	(108)

Arrow up denotes increase, arrow down denotes decrease.The members of the MMPs family are written in bold text.

## Author contributions

JC, SQ, KZ, YJ, and SL collected literatures. JC, XW, and FP analyzed literatures and summarized results. JC, SQ, and KZ drafted the manuscript. DL and CP revised the manuscript. All authors contributed to the article and approved the submitted version. 

## Glossary

**Table:**

ADAM17	A Disintegrin and A MetalloProtease Domain 17
ADSC-EVs	Adipose-derived stem cells
ADSCs	Adipose Derived Mesenchymal Stem Cells
AGEs	Advanced glycation end products
allo-mBM-MSCs	Mouse bone marrow-derived allogeneic MSCs
AMPK	Adenosine monophosphate activated protein kinase
Ang	Angiopoietin
BM	bone marrow
BM-MSCs	Bone-marrow-derived mesenchymal stem cells
CAT	Catalase
CCR7	C-C chemokine receptor type 7
circ-RNAs	circular RNAs
COL	Collagen
COX-2	Cyclooxygenase-2
CYP	Cytochrome P450
DFUs	Diabetic foot ulcers
Dll4	Delta-like 4
DM	Diabetes mellitus
E2	17β-Estradiol
ECM	Extracellular matrix
EE	Hsian-tsao extracts
EGF	Epidermal growth factor
EGFR	Epidermal growth factor receptor
EPCs	endothelial progenitor cells
ERK	Extracellular regulated protein kinases
ER-α	estrogen receptor-α
FGF	fibroblast growth factor
FOXO1	Forkhead box protein O1
GAP-43	Growth associated protein-43
G-Rb1	Ginsenoside Rb1
GSH-Px	Glutathione peroxidase
HG	High glucose
HIF-1α	hypoxia inducible factor-1α
HOXA3	Homeobox A3
IGF-1	Human insulin-like growth factor 1
IL	Interleukin
JNK	c-Jun N-terminal kinase
lncRNAs	long non-coding RNAs
LRG1	Leucine-rich α-2-glycoprotein-1
MCP-1	Monocyte chemoattractant protein-1
MDA	Malonaldehyde
MG	methylglyoxal
miRNAs	microRNAs
MMPs	Matrix metalloproteases
MSCs	Mesenchymal stem cells
NF-κB	Nuclear transcription factor-κB
NGAL	neutrophil gelatinase-associated lipocalin
NICD	Notch intracellular domain
NR1	Notoginsenoside R1
Nrf-2	Nuclear factor erythroid 2-related factor 2
PA	Patchouli alcohol
PAI-1	inhibition of Serpine1
PDC	Dendrobium Polysaccharides
RA	All-*trans*-retinoic acid
RAGE	Receptor for AGE
ROS	Reactive oxygen species
SCF	Stem cell factor
SHED	Stem cells from human exfoliated deciduous teeth
shRNA	small hairpin RNA
TACE	TNF-Alpha Converting Enzyme
T-AOC	Total antioxidant capacity
TDG	thymine-DNA glycosylase
TET2	ten-eleven translocation-2
TETILA	TET2-interacting lncRNA
TGF	Transforming growth factor
TIMPs	Tissue inhibitors of metalloproteinases
TNF	Tumor necrosis factor
TrxR1	Thioredoxin reductase 1
uPA	urokinase-type plasminogen activator
uPAR	uPA receptor
VCN-2	Vicenin-2
VEGF	Vascular endothelial growth factor
VLUs	venous leg ulcers
WEP	Hsian-tsao polysaccharides
