# Corrigendum: Targeting matrix metalloproteases in diabetic wound healing

**DOI:** 10.3389/fimmu.2023.1287048

**Published:** 2023-09-11

**Authors:** Junren Chen, Siqi Qin, Shengmeng Liu, Kexin Zhong, Yiqi Jing, Xuan Wu, Fu Peng, Dan Li, Cheng Peng

**Affiliations:** ^1^ State Key Laboratory of Southwestern Chinese Medicine Resources, School of Pharmacy, Chengdu University of Traditional Chinese Medicine, Chengdu, China; ^2^ State Key Laboratory of Quality Research in Chinese Medicine, Macau Institute for Applied Research in Medicine and Health, Macau University of Science and Technology, Taipa, Macau SAR, China; ^3^ Key Laboratory of Drug-Targeting and Drug Delivery System of the Education Ministry, Department of Pharmacology, Sichuan University, Chengdu, China; ^4^ Sichuan Engineering Laboratory for Plant-Sourced Drug and Sichuan Research Center for Drug Precision Industrial Technology, West China School of Pharmacy, Sichuan University, Chengdu, China

**Keywords:** matrix metalloproteases, diabetic wound healing, natural products, chronic inflammation, clinical research studies

In the published article, there was an error in affiliation 1. Instead of “State Key Laboratory of Characteristic Chinese Medicine Resources in Southwest China, Chengdu University of Traditional Chinese Medicine, Chengdu, China”, it should be “State Key Laboratory of Southwestern Chinese Medicine Resources, School of Pharmacy, Chengdu University of Traditional Chinese Medicine, Chengdu, China.”.

The authors apologize for this error and state that this does not change the scientific conclusions of the article in any way. The original article has been updated.

